# Artificial intelligence and digital health in the health systems of developing countries: the challenges and vision of integration in the primary health care setting

**DOI:** 10.3389/fdgth.2025.1532361

**Published:** 2025-06-26

**Authors:** Manoochehr Karami, Hayder Madlool

**Affiliations:** Department of Epidemiology, School of Public Health and Safety, Shahid Beheshti University of Medical Sciences, Tehran, Iran

**Keywords:** artificial intelligence, digital health, primary health care, health system, primary care

## Introduction

Primary health care (PHC) is the first point of contact for people to receive healthcare services in developing countries' health systems. In defining primary health care as a community-oriented approach, a wide range of essential health services, including health promotion, prevention and rehabilitation, are mentioned. Furthermore, in its basic principles, comprehensiveness, fair access, and appropriateness to the necessities and preferences of the individual, family, and society have been addressed. Importantly, PHC emphasizes well-being, people-centered care as opposed to disease-centered care, by providing services within the community. However, in low- and middle-income countries (LMICs), access to the formal health system is often limited to emergency situations, fostering an episodic model of care (1). This episodic care presents significant challenges in LMICs, primarily due to systemic issues such as inadequate infrastructure, workforce shortages, and the delivery of poor-quality services. These challenges contribute to fragmented health systems that often marginalize disadvantaged populations (2). PHC offers a strategic pathway to close these gaps and accelerate progress toward universal health coverage (UHC). According to the World Health Organization (2019), scaling up PHC in LMICs could prevent up to 60 million deaths and extend the average life expectancy by 3.7 years by 2030. Furthermore, it is estimated that 75% of the health gains required to meet the “Sustainable Development Goals” (SDGs) are achievable through strengthened PHC systems (3). However, persistent barriers remain-limited funding for preventive services, under-resourced and inadequately trained community health workers, high out-of-pocket expenditures, and fragmented insurance systems-all disproportionately affect PHC implementation in LMICs (4). This situation highlights the significant gap between the current state of LMIC health systems and the ideals of the PHC paradigm.

Since 1978, PHC has been used as the most vital way to attain the goals of “health for all”, UHC and SDGs (2, 5). The adoption of appropriate and innovative solutions to support the health of communities is an essential part of PHC-based health systems. Considering the evolution of digital health and novel technologies, health systems need to adopt such innovative tools to provide high-quality services appropriately. The vision of many countries and the recommendation of the World Health Organization are to use new technologies and innovative approaches to achieve the health system's goals and provide quality services (6). Accordingly, this article presents an overview of the current landscape of digital health and the adoption of emerging technologies-particularly artificial intelligence (AI) within primary health care systems in developing countries. Drawing on experiences and strategic guidance from the WHO, this work highlights real-world applications and policy-level insights. The article subsequently delves into the critical challenges hindering the effective integration of AI in LMICs, with particular attention given to systemic barriers, infrastructural deficits, and sociopolitical obstacles that complicate implementation efforts.

### The role of AI in primary health care

In simple language, artificial intelligence (AI) is a process in which a computer rather than human intelligence performs the tasks of health workers, such as providing screening services, training, follow-up and similar health care. Today, AI tools play a substantial role in communities' daily lives and in more specialized fields. Automated machine learning and deep learning are new fields of artificial intelligence. In the developed models in machine learning, on the basis of the written algorithm, the machine is asked to provide training in the form of a video or written media according to the question of the recipient of the service or care received. One of the most well-known new technologies used to provide health promotion services (education) is the S.A.R.A.H. system, introduced as an artificial intelligence-based system with a generative approach by the World Health Organization. This technology can be available to users 24 h a day in eight languages to provide educational and health promotion services related to various topics (7). Another example is the use of AI based on the BrewAI platform, which, by providing a simple user interface, allows non-specialists in machine learning models to enter data to predict disease outbreaks (5). In the field of preparedness and response to biological threats, the EPIWATCH platform is another example for generating epidemic warnings before identifying them with the usual health care system (8).

Recent studies have shown a growing trend in AI-driven interventions for public health, with significant improvements in diagnostics, disease prediction, and telemedicine applications. For instance, a scoping review by Abbasgholizadeh Rahimi, et al. underscores the role of AI in community-based PHC, emphasizing machine learning models for risk assessment and risk treatment adherence (9).

Despite the practical use of artificial intelligence tools in the clinical field, the use of new technologies in the field of health, specifically in primary health care, has been limited. A review of the available evidence indicates that the use of machine learning models and other artificial intelligence tools in the early identification of cancers can predict the risk of high blood pressure, the health of elderly persons, and other respiratory diseases. The findings of a systematic review revealed that the most commonly used methods for accessing new technologies for health service providers were machine learning models (9).

### Challenges of using AI in primary health care

Although the integration of digital health technologies in low- and middle-income countries (LMICs) is widely viewed as a promising advancement, significant challenges remain. Despite the possibility of enhanced access and quality of care, resistance from stakeholders who benefit from the status quo remains a key barrier. The power dynamics among patients and providers are changing in line with digital technologies, necessitating adjustments in health care policies and roles. However, disparities in access to technology create questions about equity. To certify that the advantages of digital health are justifiably distributed, effective implementation by policymakers and health care managers is vital (10).

AI in PHC presents major challenges, particularly in LMICs. A primary concern is confidentiality and data privacy, as the digitization of health records increases the risk of data breaches and unauthorized access. Ethical concerns related to AI in health care include potential violations of patient privacy, the tension among providing care and generating profit and the introduction of a third party into the patient‒doctor relationship, which modifies expectations of confidentiality and responsibility (11).

Furthermore, questions of data ownership and control remain unresolved, with governments, private companies, and individuals having competing interests. Several low-income countries also face economic constraints in adopting AI technologies, as external funding is often unsustainable, and domestic investment in digital health infrastructure is restricted. Moreover, resistance to technological change starting with stakeholders benefiting from the status quo further complicates AI implementation. An additional challenge lies in the lack of interoperability and integration with existing health systems, which can cause inefficiencies instead of improvements in healthcare delivery. Despite these barriers, AI has the potential to enhance patient outcomes, streamline workflows, and improve healthcare access if it is implemented with strong policies on governance, security, and equitable access (10).

Moreover, human aspects must be carefully considered when implementing AI in health care. This consists of assessing the skill levels, education, and computer literacy required of end users. Furthermore, the extent of the behavioral modifications necessary to build awareness and confidence in AI systems should be addressed, ensuring that employers can recognize the limitations of these technologies and correctly interpret their outputs (12).

Finally, the primary challenge in integrating AI/ML into the pursuit of the PHC vision is confirming that these technologies align with a human-centered approach. This challenge is not a natural limitation of AI/ML capabilities but rather a function of societal issues, as well as knowledge, attitudes, and behavioral intentions toward AI/ML as a tool for preventive PHC. Addressing this topic requires direct action to bridge these gaps, emphasizing value clarification and alignment with the PHC vision to unlock the worldwide benefits of AI/ML in health care (13).

### Author's vision and strategic recommendations

The successful improvement and deployment of AI in healthcare rely on the active participation of main stakeholders, including healthcare providers, patients, policymakers, and technology developers. Collaboration ensures that AI tools are user-centered, aligned with real-world primary healthcare requirements, and designed for usability and trust. Multidisciplinary corporations play a critical role in continuing regulatory and ethical standards, fostering shared responsibility and reducing resistance to AI adoption. In addition, a well-structured policy strategy is critical for addressing urgent healthcare challenges, prioritizing AI applications for early disease recognition and workload reduction while ensuring long-term improvements in interoperability, algorithm refinement, and equitable access. Ethical concerns, such as transparency and accountability, must be essential to regulatory frameworks to maximize AI benefits while mitigating threats.

In alignment with the World Health Organization's vision of leveraging innovative technologies to develop access, improve the quality of healthcare and advance community health, public health authorities need to establish the necessary infrastructure for AI integration. Digital health should be recognized as a top priority for developing countries, with AI applications implemented in an ethical, safe, secure, sustainable and equitable manner. Integration efforts should adhere to principles of transparency, accessibility, scalability, repeatability, confidentiality, and trust, ensuring seamless data sharing across relevant systems (14).

In this context, public health authorities in developing countries must mobilize and engage key stakeholders to develop an operational plan that aligns with the World Health Organization's proposed solutions for short-, medium-, and long-term implementation. Strengthening healthcare networks through the integration of new technologies is crucial for addressing current challenges, delivering high-quality, people-centered care, and ensuring effective coverage.

As practical recommendations, policymakers and public health authorities of health systems should formulate strategies and implement feasible interventions for integrating digital health into PHC systems. Health system policymakers should ensure that these interventions and recommendations provide a flexible, locally adapted roadmap at both the national and subnational levels. This approach would enable the systematic piloting, practice, and full integration of digital health solutions into PHC centers.

One of the key challenges in adopting AI tools is the complexity of the algorithms and models, which can hinder their use by healthcare workers. To effectively integrate artificial intelligence into PHC centers, comprehensive policies are essential to support the translation of existing knowledge into practice. This includes ensuring cybersecurity and developing locally adapted models for optimal use. Moreover, the responsible and ethical use of available data, alongside continuous evaluation of AI-driven healthcare systems, is essential to maintaining trust, effectiveness and sustainability in digital health transformation.
